# Temporal Consistency and Averaging Sensitivity of Released Smart-Insole Gait Summaries in Participants with and without Knee Osteoarthritis

**DOI:** 10.3390/s26206519

**Published:** 2026-10-14

**Authors:** Mi-Ae Yang, Kang-Su Ha

**Affiliations:** 1 Department of Science and Technology Convergence, Graduate School, Chosun University, Gwangju 61452, Republic of Korea; 2 Department of Psychiatry, Rainbow Hospital, Gwangju 61924, Republic of Korea; 3 College of Medicine, Chosun University, Gwangju 61452, Republic of Korea

**Keywords:** coefficient of variation, gait monitoring, intraclass correlation coefficient, knee osteoarthritis, measurement agreement, plantar pressure, repeated observations, smart insole, temporal consistency, wearable sensors

## Abstract

Repeated smart-insole outputs may support longitudinal gait research, but consistency of processed summaries does not establish device accuracy or a clinical monitoring schedule. We analyzed 10 released observations per participant from AI Hub dataset 71966, retaining 178 participants (89 provider-labeled normal and 89 knee osteoarthritis) after exclusion of a previously reported opposing-label duplicate pair. Analyses combined absolute-agreement intraclass correlations (ICCs), participant-bootstrap intervals, residual variability, nonoverlapping 2-, 3-, and 5-observation averages, and exploratory adjusted coefficient-of-variation (CV) models. Single-observation ICCs were 0.827–0.844 for midfoot/rearfoot pressure, 0.663–0.727 for stride length, speed, and forefoot pressure, 0.398 for distance, and approximately 0.014 for balance. Five-observation empirical ICCs were lower than Spearman–Brown predictions for every non-balance endpoint; gait speed yielded 0.887 versus 0.927 and left stride length 0.870 versus 0.908. Left-balance residual SD was 2.388 in the released scale despite low median CV. Linear-programming median regression and solution-choice sensitivity replaced warning-prone bootstrap fitting; the maximum change of a nominal interval endpoint from its reference was 0.0996 CV percentage points. Calendar dates, wear time, and criterion measurements were unavailable. These results characterize release-specific temporal consistency and qualify model-based averaging projections; they do not validate calendar-duration prescriptions, clinical change thresholds, or the underlying device.

## 1. Introduction

Wearable sensing can extend gait assessment beyond a single laboratory visit. Knee osteoarthritis (OA) can affect gait through pain, altered loading, and compensatory movement, while demographic characteristics and walking speed also influence the measured features [1,2,3,4,5]. Standardized instrumented walkways provide useful evidence about reproducibility of spatiotemporal measurements [6,7]. These findings motivate repeated wearable measurements but do not establish that any particular daily summary, population, or sampling schedule will be reliable.

Validation and test–retest studies of insole systems show parameter-specific performance under defined acquisition conditions [8,9,10,11]. Insole-derived signatures have also been studied for knee-arthropathy identification [12], and wearable assessment at home has been evaluated in people with knee OA [13]. Device agreement, classification performance, and consistency of repeated processed outputs are different measurement questions. A daily summary can be reproducible yet biased relative to a reference instrument, or can fluctuate because exposure and behavior change rather than because a sensor is inaccurate.

The AI Hub Musculoskeletal Disease Biosignal Data release supplies 10 selected smart-insole observations per participant [14]. Our related Bioengineering article used the same resource for release-integrity assessment and subject-level OA classification across gait-mat and multisensor configurations [15]. The present contribution is limited and distinct: (i) characterization of temporal consistency and absolute variability of the distributed smart-insole summaries; (ii) an internal empirical check of averaging projections within the available observations; and (iii) transparent uncertainty and numerical-sensitivity assessment of exploratory CV contrasts. No classification model, ROC/AUPRC result, gait-mat/IMU fusion result, provider-model reproduction result, table, or figure from the earlier article is reused. Its duplicate-pair finding informs cohort exclusion rather than a new integrity outcome.

Prior wearable studies show that the number and spacing of observations can affect reliability [16,17]. Real-world validation work emphasizes acquisition context and processing pipelines [18], and between-day results can differ even among outputs from the same device class [19]. Reliability is therefore a property of a specified measure, cohort, and sampling scheme, whereas agreement concerns the magnitude of differences [20,21]. Here, the 10 observations are an availability constraint, not a prospectively selected monitoring duration. We aim to describe release-specific temporal consistency and assess the sensitivity of model-based averaging estimates, without prescribing population-specific or real-world monitoring windows.

## 2. Materials and Methods

### 2.1. Study Design and Analysis Provenance

This secondary measurement study used deidentified labeling files accessed through AI Hub under its access rules and terms of use. As stated in the submitted manuscript, the original analysis specification was formalized on 2 August 2026 after feasibility exploration and was not prospectively registered. It specified the original endpoints, ANOVA-based ICCs, participant bootstrap, reliability targets, multiplicity procedure, and duplicate-pair exclusion. The resulting estimates are not confirmatory clinical findings.

In response to peer review, analyses conducted on 28 September 2026 added distributional checks, balance redundancy assessment, BCa ICC intervals, residual-repeatability summaries, empirical averaging comparisons, released-index diagnostics, and participant-bootstrap median regression. Numerical optimization and solution-choice audits were then added to the regression sensitivity analysis. These additions are explicitly post-review. We retain the original aggregate results for traceability and distinguish them from the repaired regression estimates; the original analysis specification has not been retroactively described as including the new analyses.

### 2.2. Source, Cohort, and Measurement Scope

AI Hub dataset 71966, version 1.1, is the Musculoskeletal Disease Biosignal Data resource [14]. Its documentation describes 15 days of smart-insole collection during daily life, with 10 selected observations distributed in the structured release. The selection algorithm, full 15-observation series, calendar-date mapping, exact wear time, step count, and activity context are unavailable. We therefore use “released observation” or “released index” for day_1 through day_10; consecutive calendar days, exposure duration, and sampling regularity cannot be verified.

The Training and Validation smart-insole partitions contain 180 participants, 90 in each provider-labeled group. SUBJ_247 and SUBJ_253 were previously identified as an exactly duplicated smart-insole payload with opposing labels [15]. Their exclusion leaves 178 participants, 89 per group, and 1780 participant-observations. The original sensitivity analysis restored the pair. Source group labels were used as supplied; clinical severity and independent diagnostic adjudication were not available.

Twelve continuous fields were retained for descriptive profiling: bilateral balance and stride length, gait distance, gait speed, and bilateral forefoot, midfoot, and rearfoot pressure. All scale-dependent variability summaries use the values and scale in the distributed files; calibrated pressure units or a correspondence to clinical change thresholds are not assumed. Raw pressure waveforms, step-level trajectories, an independent reference system, and calibration records were unavailable. Bilateral foot_angle codes 0/1/2 were analyzed as nominal provider-defined labels, not interval-scale angular measurements.

The local preflight checked participant/index uniqueness, expected indices 1–10, class-label consistency within participant, numerical finiteness, and availability of age, sex, and BMI. Missingness and distribution summaries were computed for each field. Values outside Q1 − 1.5 × IQR or Q3 + 1.5 × IQR were flagged descriptively, not removed. No imputation, winsorization, or outcome-driven participant exclusion was used. Correlation and the maximum absolute departure of left-plus-right balance from 100 quantified near-complementarity; right balance remained descriptive but was excluded from the 11-endpoint regression and temporal-diagnostic set. This does not imply that the remaining endpoints are statistically independent.

### 2.3. Statistical Analysis and Sensitivity Analyses

#### 2.3.1. ICCs and Bootstrap Uncertainty

For each continuous field, the 178 × 10 participant-by-occasion matrix was analyzed using a two-way random-effects, absolute-agreement ICC for one observation, ICC(A,1), and the model-based mean of 10 observations, ICC(A,10) [20]. With *n* participants, *k* occasions, and subject, occasion, and residual mean squares MSR, MSC, and MSE, respectively, ICC(A,1) = (MSR − MSE)/[MSR + (*k* − 1)MSE + *k*(MSC − MSE)/*n*], and ICC(A,*k*) = (MSR − MSE)/[MSR + (MSC − MSE)/*n*]. Method-of-moments subject and occasion components are (MSR − MSE)/*k* and (MSC − MSE)/*n*; the residual component is MSE.

The ICC estimates were not truncated at zero. The original variance-percentage table, however, used nonnegative parts of subject/occasion components before normalization; this display convention is identified in Supplementary Table S2 and is not a constrained REML fit. Percentile intervals from the original analysis are retained for comparison. The post-review ICC(A,1) sensitivity analysis used bias-corrected and accelerated (BCa) 95% intervals with 10,000 participant resamples, keeping all observations of each selected participant together, and leave-one-participant-out jackknife acceleration. BCa results were compared with percentile limits from the same resamples. Negative limits indicate uncertainty of the unconstrained estimator near zero, not negative physical variance. BCa does not itself establish boundary coverage; no coverage simulation or REML benchmark was performed.

#### 2.3.2. Absolute Variability and Agreement Limits of Interpretation

To complement ICCs, we report residual SD = √MSE, the residual SEM used by the executed code, and nominal RC95 = 1.96√2 × residual SD. The latter has the conventional MDC95 form and equals the code’s repeatability-coefficient output; these are not two independent results. This residual SEM excludes the common occasion variance and must not be relabeled as total absolute-agreement SEM. The residual also mixes unexplained biological, exposure, processing, and measurement variation, so neither quantity isolates device noise or establishes a clinically validated minimum detectable change [21].

For an additional descriptive agreement check, differences between adjacent released indices were pooled across participants. Their mean and mean ± 1.96 sample SD are reported as descriptive adjacent-difference bands. Each person contributes nine correlated differences, yielding 1602 differences in total. These summaries are not independent-pair or repeated-measures Bland–Altman limits with validated coverage, and do not compare the insole against a criterion instrument. No clinical acceptability threshold or inferential uncertainty for the bands is claimed.

#### 2.3.3. Model Projections and Empirical Averaging

The Spearman–Brown relation R(m) = mr/[1 + (m − 1)r], with r = ICC(A,1), describes model-based averaging under a stable between-participant component and exchangeable repeated-error structure [22]. For 0 < r < 1, the smallest integer m reaching target T is ceil{T(1 − r)/[r(1 − T)]}, calculated before display rounding. Targets 0.80 and 0.90 are descriptive, not clinical decision thresholds. Every count is hypothesis-generating; counts above 10 additionally extrapolate outside the released observations. For near-zero balance ICCs with intervals spanning zero, a defensible finite requirement is not reported (NR). A small positive point estimate permits a finite algebraic projection, but that projection is unstable as r approaches zero and does not support the former “>30 days” display as a useful requirement.

For empirical comparison, each participant’s values were averaged into nonoverlapping blocks of m = 2, 3, or 5 released indices. These yield five two-observation blocks (indices 1–10), three three-observation blocks (indices 1–9; index 10 unused), and two five-observation blocks (indices 1–5 and 6–10). ICC(A,1) across block means was compared descriptively with R(m). Block nonoverlap does not establish temporal independence; all comparisons use the same cohort, and no interval or hypothesis test for empirical-minus-predicted differences was calculated. Ten available observations yield only one disjoint 10-observation mean per participant, so no independent empirical repeatability comparison of two such means is possible. ICC(A,10) is model-based and algebraically equals R(10), not a separate validation of that projection.

#### 2.3.4. Group Contrasts and Regression Numerical Sensitivity

Original group-specific ICCs are descriptive. Participant CV was 100 × sample SD/absolute mean over the 10 observations. The original conditional-median regression used provider class, age, sex, BMI, and an intercept [23], with fitted-model uncertainty and Benjamini–Hochberg adjustment of 12 class-effect tests [24]. These original results are retained as historical comparators in Supplementary Table S5, not treated as the new bootstrap inference. Right balance is omitted from the post-review 11-endpoint regression set to avoid interpreting nearly duplicated balance information twice.

The first participant-bootstrap implementation produced iteration-limit warnings in iteratively reweighted least-squares fitting. We therefore solved the same unpenalized median-regression objective using SciPy linear programming and the HiGHS dual-simplex optimizer, with centered/scaled age, BMI, and outcome for numerical computation and coefficients returned to CV percentage points. All 1000 participant-pairs resamples per endpoint were retained, with covariate and outcome rows sampled together. Optimality diagnostics were inspected rather than counting warning-returned coefficients as convergence successes. The resulting 95% percentile intervals and bootstrap SDs are exploratory and unadjusted for multiplicity. We do not interpret the earlier bootstrap sign-tail proportions or their BH transforms as calibrated null-hypothesis *p* values or FDR discoveries.

The designated dual-simplex solution remained the reference. For each full-data and bootstrap fit, the minimum and maximum class coefficient compatible with scaled quantile loss ≤ L* + 10^−10^ max(1,L*) were evaluated, where L* is the reference optimum. A coefficient-range screen of 0.0001 CV percentage points was numerical only. Fits exceeding it were additionally evaluated with a 10^−8^ loss budget. Quantiles of coefficient bounds describe possible movement of percentile-interval endpoints; they are numerical envelopes, not replacement confidence intervals or tests. No reference coefficient, resample, or seed was chosen according to significance.

#### 2.3.5. Released-Index and Distributional Sensitivity

Within-person linear trends were estimated by regressing participant-centered values on centered released index with participant-cluster-robust standard errors; the 11 trend *p* values were BH-adjusted. Adjacent-index correlations were calculated from participant-centered 10-observation sequences, with percentile intervals from 2000 participant resamples. These are finite-series, released-index diagnostics, not estimates of calendar-day autocorrelation with verified spacing. Centering each short sequence can itself induce dependence, so negative correlations and absence of significant trends cannot establish the absence or direction of physiological serial dependence. Weekday, seasonal, wear-time, and adaptation effects could not be separated; no AR(1)-adjusted projection was fitted.

Gait-distance sensitivity compared the original scale with log(1 + distance), retaining all observations. ICCs on the transformed scale answer a different scale-specific question and were not substituted for the raw-scale analysis. The participant-based resampling design preserves dependence within each person rather than resampling observations as independent rows.

#### 2.3.6. Categorical Stability, Provenance, and Software

Foot-angle labels were summarized by Fleiss’ kappa [25], the percentage unchanged over all 10 observations, and within-participant modal-category agreement. Label persistence does not quantify angular drift within a category, proximity to a boundary, or stability of a clinical classification. The previously reported duplicate-inclusion sensitivity is retained separately. Source-data and analytical artifacts are distinguished in the Supplementary Materials.

The post-review base random seed was 20260902. ICC seeds were base + j for the 12-field order in Supplementary Table S7; temporal-diagnostic seeds were base + 100 + j and regression seeds base + 200 + j for the 11-endpoint order in Supplementary File S2, with j starting at zero. Post-review computations used Python 3.13.5, NumPy 2.3.5, pandas 2.2.3, SciPy 1.18.0, statsmodels 0.14.6, and Matplotlib 3.10.8. Code and aggregate outputs are supplied in Supplementary File S2; author-local source files and participant-level matrices are not redistributed. These materials distinguish numerical checks from statistical and clinical validation.

### 2.4. Ethics and Source-Data Governance

No participants were newly recruited or contacted, and no intervention, re-identification attempt, or patient-directed decision was performed. The source documentation reports ethics review and consent procedures for the original collection. This analysis used deidentified secondary files under the platform’s access conditions. No new consent was obtained by the authors. AI-assisted preparation is disclosed in the Acknowledgments; the authors remain responsible for analytical choices, verification, and interpretation.

## 3. Results

### 3.1. Cohort, Completeness, and Distribution

The primary cohort comprised 178 participants and 1780 observations, with exactly 10 indices per participant and no duplicate participant/index rows in the local preflight. Required continuous fields had no missing values in this retained release. This completeness does not recover the five unprovided observations from the original collection. The normal group’s age was 50.0 ± 8.7 years, 59/89 participants were women (66.3%), and BMI was 23.0 ± 3.4 kg/m^2^; corresponding OA values were 58.6 ± 9.7 years, 73/89 (82.0%), and 24.4 ± 3.7 kg/m^2^ (Supplementary Table S1).

Left/right balance correlation was −0.999854, with maximum |left + right − 100| = 1; thus the fields were nearly, not exactly, complementary. Balance Q1, median, and Q3 were all 50, making the IQR zero and flagging 681 observations per side by the descriptive fence rule. These flags were not treated as errors or exclusions. Left balance ranged from 29 to 69. Gait distance ranged from 22 to 10,160 and had skewness 3.205, with 102/1780 observations (5.73%) outside the IQR fences. All values were retained (Supplementary Table S6).

### 3.2. ICC Profile and Absolute Variability

**Figure 1:**
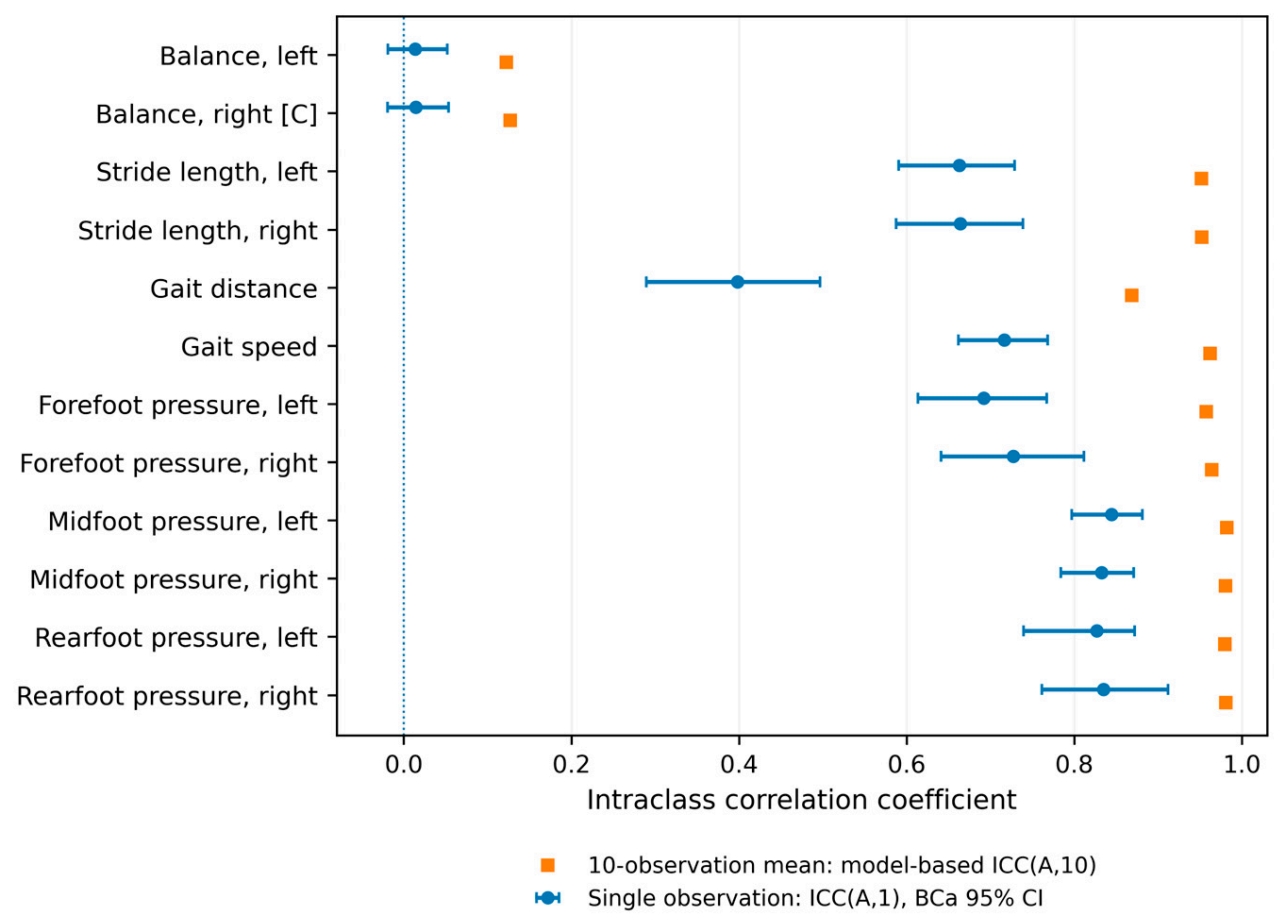
Pooled single-observation ICC(A,1) with BCa 95% confidence intervals, and model-based ICC(A,10) point estimates. The 10-observation estimates are algebraic mean-reliability quantities, not independent empirical repeatability estimates; their original percentile intervals are in Supplementary Table S2. [C] denotes the near-complementary right-balance field, retained descriptively.

Pooled ICCs are summarized in Table 1 and Figure 1. Single-observation ICC(A,1) was 0.827–0.844 for midfoot/rearfoot pressure and 0.663–0.727 for stride length, speed, and forefoot pressure. Gait distance was 0.398 (BCa 95% CI 0.289–0.496). Balance was approximately 0.014 on each side, with BCa intervals spanning zero. Percentile-versus-BCa comparisons are supplied in Supplementary Table S7. The model-based ICC(A,10) exceeded 0.95 for stride length, speed, and pressure summaries, but was 0.869 for distance and approximately 0.12 for balance; this transformation is not an independent empirical result.

Table 2 provides scale-dependent variability. Left-balance residual SD was 2.388 and nominal RC95 was 6.618; the adjacent-difference mean was 0.021, with a descriptive band of −6.621 to 6.663. Thus the low median balance CV of about 1% does not imply uniformly tiny differences, particularly in this concentrated but long-tailed distribution. Gait-speed residual SD was 0.398 and distance residual SD was 513.679 in their respective released scales. These values describe unresolved variability of processed outputs, not isolated device error or validated clinical change thresholds. The right balance and additional scale summaries are retained in Supplementary Table S8.

### 3.3. Empirical Averaging and Projection Limits

**Figure 2:**
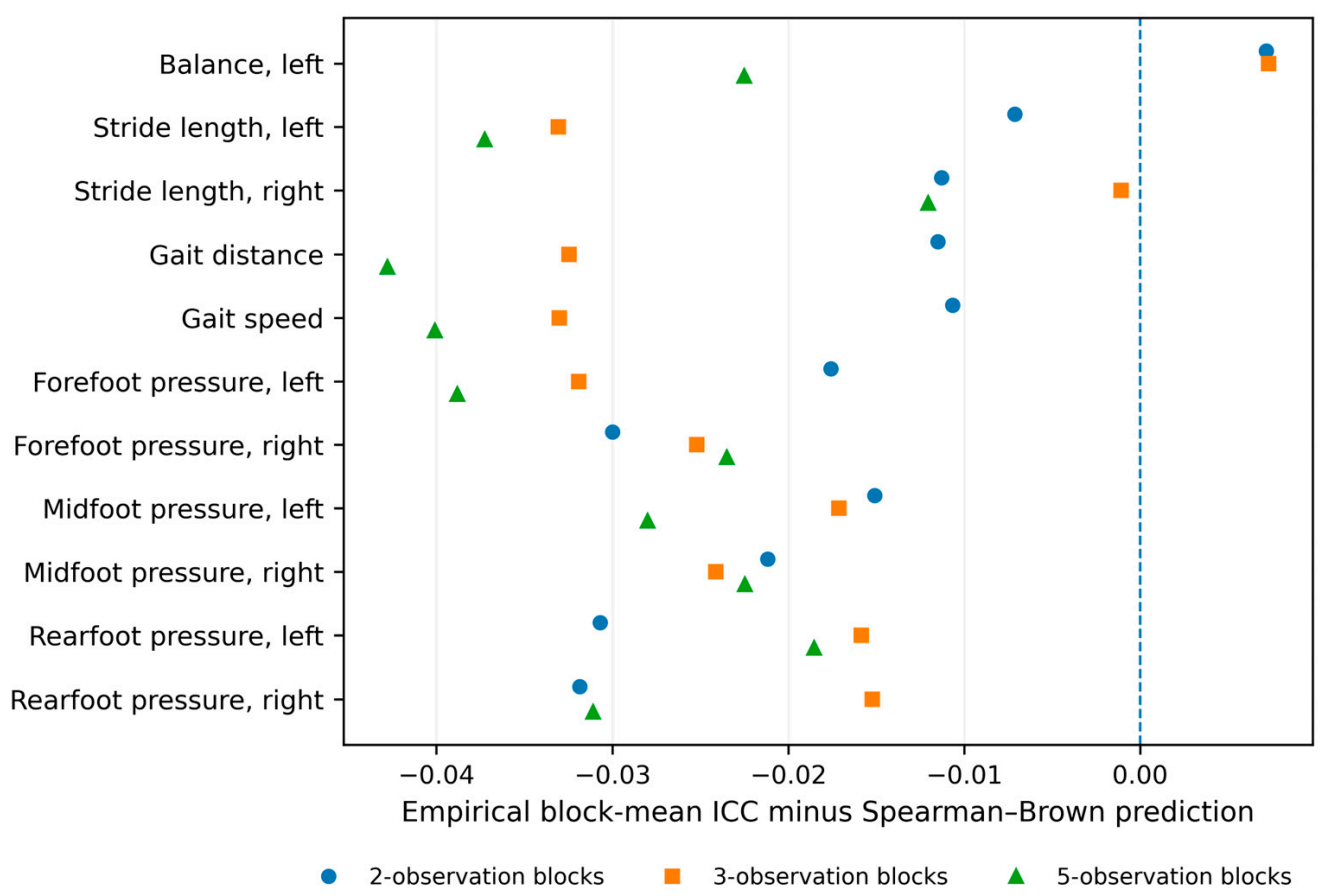
Descriptive differences between empirical nonoverlapping block-mean ICC(A,1) and the Spearman–Brown prediction, using two, three, or five released observations per block. Negative values indicate a lower empirical point estimate. The three-observation comparison uses indices 1–9 only.The dashed line marks zero. Within-cohort differences have no confidence intervals; nonoverlap does not establish temporal independence.

Table 3 and Figure 2 compare observed block-mean ICCs with Spearman–Brown predictions. All 30 comparisons for the 10 non-balance endpoints had lower empirical point estimates. For five-observation means, left stride length was 0.870 versus a prediction of 0.908; gait speed was 0.887 versus 0.927; and distance was 0.725 versus 0.768. Several predictions above 0.90 therefore did not correspond to empirical block-mean estimates above 0.90. These are descriptive differences, not a statistical demonstration of predictive bias across populations.

**Figure 3:**
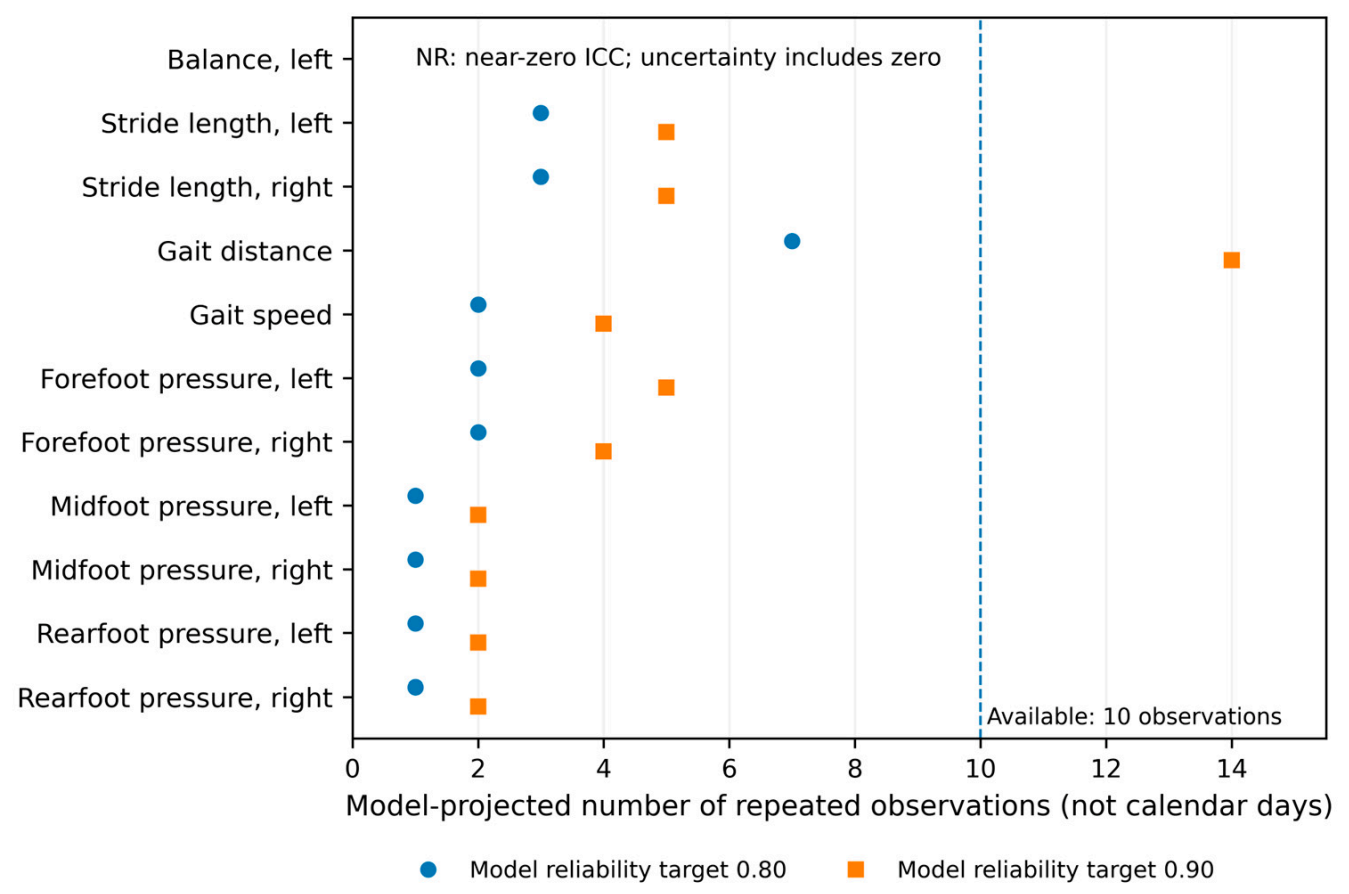
Hypothesis-generating model projections for the number of repeated observations associated with reliability targets 0.80 and 0.90. The dashed line marks the 10-observation availability boundary, not verified calendar duration. The 14-observation distance projection is extrapolative. NR indicates an unstable finite requirement for near-zero balance ICC with uncertainty spanning zero; no >30-day truncation is used.

The model-projected counts are shown in Figure 3. At target 0.90, point projections were two observations for midfoot/rearfoot pressure, four for speed and right forefoot pressure, and five for bilateral stride length and left forefoot pressure. Distance projected to seven observations at 0.80 and 14 at 0.90. The 14-observation estimate exceeds the available 10-observation range; all counts, including those inside that range, remain hypothetical model projections rather than prescribed calendar durations. Finite balance requirements are not reported because near-zero estimates and uncertainty spanning zero make the inverse projection unstable.

### 3.4. Exploratory Group Contrasts and Numerical Sensitivity

Original group-specific ICCs remain descriptive (Supplementary Table S3). For example, left stride-length ICC(A,1) was 0.772 in the normal group and 0.532 in the OA group, without a formal between-ICC test. Such contrasts do not establish population-specific observation schedules.

**Figure 4:**
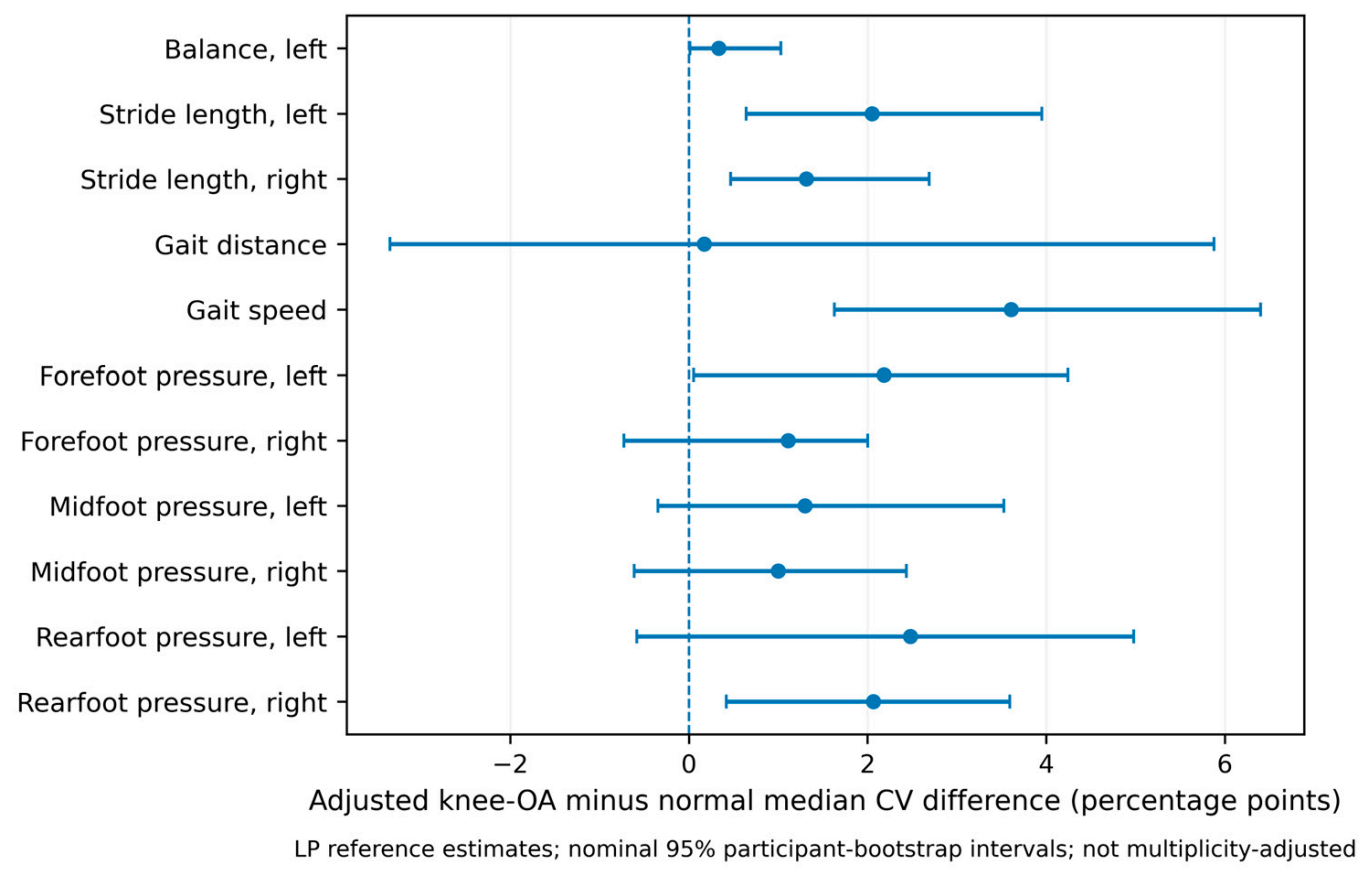
Linear-programming reference coefficients for adjusted knee-OA-minus-normal conditional-median CV differences, with nominal 95% participant-pairs bootstrap intervals (1000 draws per endpoint). Age, sex, and BMI are included as covariates. Intervals are exploratory and not multiplicity-adjusted; no significance stars or FDR discoveries are assigned. Numerical solution-choice envelopes are reported separately in Supplementary Table S13. The dots show point estimates, the horizontal lines show nominal 95% intervals, and the dashed vertical line denotes zero adjusted difference.

The repaired linear-programming regression estimates and nominal participant-bootstrap intervals appear in Table 4 and Figure 4. The class coefficient for gait-speed CV was +3.610 percentage points (95% interval 1.628–6.400), and that for left rearfoot-pressure CV was +2.481 (−0.584–4.979). The latter interval includes zero, unlike the original fitted-model interval. The repaired left forefoot-pressure coefficient was 2.183419, compared with the earlier iterative estimate 2.190083. These explicit changes are documented in Supplementary Tables S5 and S12; the original statement of six FDR-significant class effects is not carried forward as a claim about the new bootstrap analysis.

All 11,000 resampling-index hashes matched the reference regression audit. The maximum full-data coefficient range under the tight numerical loss budget was 0.00000365 CV percentage points; none of the 11 full-data ranges exceeded the screen. Among bootstrap fits, 1971/11,000 (17.9%) exceeded it, and 199 showed tolerance-sensitive endpoints in the conditionally checked subset. The widest individual-draw coefficient range was 2.3631 percentage points, not a change in an interval endpoint. The maximum span of possible lower and upper percentile endpoints was 0.1311 and 0.0995, respectively; the maximum absolute departure from a reference interval endpoint was 0.0996. Zero-inclusion did not change for any endpoint within the audited tight-budget choices. These numerical observations do not establish statistical coverage, FDR control, or clinical equivalence. The automatic status SOLUTION_TOLERANCE_REVIEW_REQUIRED is retained and qualified by this sensitivity report (Supplementary Table S13).

### 3.5. Released-Index, Scale, and Categorical Sensitivity

No linear released-index trend had a BH-adjusted q value below 0.05 across the 11 endpoints (minimum q = 0.301). Participant-centered adjacent-index correlations ranged from −0.127 for left balance to +0.147 for right rearfoot pressure. Several nominal intervals excluded zero, but sequence centering, selected indices, and unknown spacing prevent interpretation as validated calendar-time dependence or independence (Supplementary Table S10).

On the log(1 + distance) scale, distance ICC(A,1) was 0.472 and ICC(A,10) 0.899, versus 0.398 and 0.869 on the original scale; skewness changed from 3.205 to −1.028. This illustrates scale sensitivity rather than normalization or validation of an observation-count requirement (Supplementary Table S11).

Foot-angle labels were identical across all 10 observations for 94.4% of participants on the left and 89.3% on the right. Mean modal agreement was 97.9% and 97.1%, and Fleiss’ kappa was 0.694 and 0.625. These findings describe categorical label persistence only (Supplementary Table S14). Restoring the previously excluded pair changed the original pooled ICC(A,1) by at most approximately 0.00245 (Supplementary Table S4); this sensitivity does not resolve unobserved selection or device-validity concerns.

## 4. Discussion

### 4.1. Principal Findings and Revised Scope

This study describes temporal consistency of processed smart-insole outputs within one selected AI Hub release. The contrast between high pressure-summary ICCs, moderate stride/speed ICCs, and near-zero balance ICCs is retained, but the interpretation has changed: model-based averaging gains do not establish the number of calendar days a participant should be monitored. Empirical block-mean estimates were lower than model predictions for every non-balance comparison examined. In particular, five-observation estimates for gait speed and left stride length did not reach 0.90 despite predictions exceeding that value. The 10-observation ICC is a model transformation, not an independent replication.

### 4.2. Relation to Published Averaging Studies

Published estimates provide context rather than external validation. Buckley et al. analyzed 197 residential-care participants with at least three monitored days and reported approximately two to five days, depending on walking outcome and care level [16]. Hilden et al. analyzed at least 60 valid days from 69 participants and reported six days for light activity, eight for sedentary time, and ten for moderate-to-vigorous activity to reach reliability 0.80 [17]. Those populations, device locations, outcomes, valid-day rules, and designs differ from the present selected insole release. Its algebraic 0.90 projections of two to five observations for many endpoints, and 14 for distance, are therefore not interchangeable with those published day counts. Our internal block comparison, rather than numerical similarity to another paper, directly examines the adequacy of this release’s averaging model.

The present estimates also differ in purpose from criterion or test–retest validation of a device [8,9,10,11,13,18]. Such studies can evaluate reference-system agreement and standardized tasks. Here, the absence of a criterion instrument and exposure metadata precludes equivalent validation. The results should not be used to claim that a particular pressure sensor is accurate, that a four-observation average tracks pain or function, or that healthy and OA populations require different real-world schedules.

### 4.3. What the Variance Components Can and Cannot Identify

Near-complementary balance fields should not count as two independent findings. Their near-zero ICCs and small median CVs are compatible with little stable between-person separation, but that is not proof that cohort homogeneity alone explains the result. The retained distribution includes larger deviations, and pooled residual SD reflects these tails. Quantization, preprocessing, restricted range, exposure differences, device limitations, and biological variation cannot be separated with the available summaries. The normalized variance percentages and distributional diagnostics make this uncertainty explicit rather than establishing a hardware mechanism.

Adding residual SEM and an RC95/MDC95-form multiplier improves scale transparency but does not turn the outputs into clinically usable change thresholds. Likewise, adjacent-difference bands are descriptive because pairs overlap within participants and there is no criterion instrument. A formal repeated-measures agreement analysis with justified error structure, and an external clinical anchor would be needed for stronger interpretation. No REML or generalizability-theory benchmarking was performed; the ANOVA estimand, component-display convention, and empirical averaging check are reported rather than presenting those alternatives as completed analyses.

### 4.4. Regression Uncertainty and Solution Choice

The revised group analysis emphasizes estimation and sensitivity, not a list of discoveries. Iteration-limit warnings in the first bootstrap prompted a solver change for the same median-regression objective, not a change in participants, outcomes, or resampling to favor significance. Reporting the LP reference estimates, the original fitted-model comparators, and numerical coefficient-choice ranges allows the changes to be traced. Some resamples admit materially wider near-optimal coefficient ranges even though full-data coefficients are stable under the numerical screen.

The limited movement of percentile endpoints within the audited ranges is a numerical finding only. It neither proves uniqueness nor validates bootstrap coverage in this sample. The looser tolerance was investigated only for screen-positive draws, and the corresponding check is not a complete loose-budget envelope. The class-effect intervals are unadjusted exploratory estimates; the prior sign-tail *p* values and FDR labels are not reused. Demographic imbalance and absent clinical/exposure covariates further prevent a causal interpretation of OA-associated variability.

### 4.5. Limitations and Reproducibility

The main limitations are selected rather than complete observation series; unknown selection rules, calendar dates, wear time, steps, and activity context; processed rather than raw signals; no criterion instrument or clinical outcome anchor; unmatched groups; and a single national data resource. The release cannot support population-specific monitoring recommendations. Released-index diagnostics do not recover weekday or adaptation effects, and lack of a significant linear trend does not verify exchangeability. Nonoverlapping block means remain dependent observations from the same selected series. The three-observation comparison omits index 10, and the five-observation comparison has only two blocks per participant.

All post-review additions are exploratory. BCa intervals are a sensitivity analysis, not proof of boundary coverage; absolute-repeatability summaries have no validated clinical threshold; and empirical averaging differences have no reported confidence intervals. A complete alternative variance-component benchmark and a coverage study were not performed. Code, seeds, recorded numerical audits, and aggregate results are supplied, but participant matrices and original JSON files are not redistributed. Independent raw-data reproduction therefore requires authorized access to AI Hub; the supplementary archive is not a stand-alone replacement for the source data. Neither successful local execution nor aggregate arithmetic checks establish clinical validity.

## 5. Conclusions

Across 10 selected released observations per participant, smart-insole summaries show endpoint-specific temporal consistency and absolute variability. Empirical averaging comparisons qualify the higher reliability implied by model-based projections, while balance and regression results require explicit distributional and numerical-sensitivity interpretation. These data support bounded, hypothesis-generating measurement research on the distributed summaries. They do not establish an optimal monitoring duration in calendar days, population-specific acquisition prescriptions, criterion validity of the device, or clinically validated change thresholds.

## Figures and Tables

**Table 1:** Pooled reliability of the released summaries (178 participants; 10 observations each).

Summary	ICC(A,1)	BCa 95% CI for ICC(A,1)	Model ICC(A,10)
Balance, left	0.014	−0.019–0.052	0.123
Balance, right [C]	0.014	−0.019–0.053	0.127
Stride length, left	0.663	0.590–0.729	0.952
Stride length, right	0.664	0.587–0.739	0.952
Gait distance	0.398	0.289–0.496	0.869
Gait speed	0.716	0.662–0.768	0.962
Forefoot pressure, left	0.692	0.613–0.767	0.957
Forefoot pressure, right	0.727	0.641–0.812	0.964
Midfoot pressure, left	0.844	0.797–0.881	0.982
Midfoot pressure, right	0.833	0.784–0.871	0.980
Rearfoot pressure, left	0.827	0.739–0.872	0.979
Rearfoot pressure, right	0.835	0.761–0.912	0.981

Note. [C] indicates the descriptive near-complementary right-balance field. The ICC(A,10) values are model-based and algebraically equivalent to Spearman–Brown averaging, not an empirical comparison of independent 10-observation means. Original percentile intervals are retained in Supplementary Tables S2 and S7.

**Table 2:** Residual variability and descriptive adjacent-index differences.

Summary	Residual SD	Nominal RC95	Mean Difference	Descriptive Difference Band
Balance, left	2.388	6.618	0.021	−6.621–6.663
Stride length, left	8.655	23.990	0.096	−22.882–23.074
Stride length, right	8.467	23.470	−0.104	−22.472–22.265
Gait distance	513.679	1423.844	−7.782	−1400.278–1384.715
Gait speed	0.398	1.103	0.000	−1.035–1.035
Forefoot pressure, left	4.341	12.032	−0.099	−11.508–11.311
Forefoot pressure, right	3.999	11.085	0.022	−10.103–10.147
Midfoot pressure, left	2.706	7.500	0.027	−6.714–6.769
Midfoot pressure, right	2.779	7.703	−0.005	−6.769–6.759
Rearfoot pressure, left	3.273	9.072	0.071	−7.941–8.083
Rearfoot pressure, right	3.091	8.569	−0.017	−7.410–7.376

Note. All quantities use each field’s released scale; cross-field magnitudes are not directly comparable. Residual SD = √MSE (residual SEM only); nominal RC95 = 1.96√2 × residual SD, identical to the MDC95-form output. Bands are mean difference ± 1.96 SD from 1602 correlated adjacent differences, not coverage-validated Bland–Altman limits or clinical change thresholds.

**Table 3:** Predicted versus empirical reliability of nonoverlapping observation-block means.

Summary	2 Observations P/E	3 Observations P/E	5 Observations P/E
Balance, left	0.027/0.034	0.040/0.048	0.065/0.043
Stride length, left	0.797/0.790	0.855/0.822	0.908/0.870
Stride length, right	0.798/0.787	0.856/0.855	0.908/0.896
Gait distance	0.569/0.558	0.665/0.632	0.768/0.725
Gait speed	0.835/0.824	0.883/0.850	0.927/0.887
Forefoot pressure, left	0.818/0.800	0.871/0.839	0.918/0.879
Forefoot pressure, right	0.842/0.812	0.889/0.864	0.930/0.907
Midfoot pressure, left	0.916/0.901	0.942/0.925	0.964/0.936
Midfoot pressure, right	0.909/0.888	0.937/0.913	0.961/0.939
Rearfoot pressure, left	0.905/0.875	0.935/0.919	0.960/0.941
Rearfoot pressure, right	0.910/0.878	0.938/0.923	0.962/0.931

Note. P = Spearman–Brown prediction; E = empirical ICC(A,1) of block means. Five, three, and two blocks are available for m = 2, 3, and 5, respectively. The m = 3 analysis omits index 10. No confidence interval or formal test for P–E differences was computed. Right balance is omitted as near-complementary; the remaining endpoints are not assumed statistically independent.

**Table 4:** Exploratory conditional-median CV contrasts using the repaired LP estimator.

Summary	Normal Median CV (%)	OA Median CV (%)	Adjusted Difference (pp)	Nominal Bootstrap 95% CI (pp)
Balance, left	1.04	1.39	+0.335	0.014–1.030
Stride length, left	3.03	5.42	+2.050	0.643–3.954
Stride length, right	3.43	4.92	+1.317	0.468–2.691
Gait distance	12.61	14.47	+0.172	−3.345–5.880
Gait speed	5.74	9.81	+3.610	1.628–6.400
Forefoot pressure, left	5.28	6.87	+2.183	0.052–4.243
Forefoot pressure, right	5.06	5.97	+1.111	−0.727–2.004
Midfoot pressure, left	4.35	6.09	+1.301	−0.346–3.526
Midfoot pressure, right	4.31	5.37	+1.000	−0.614–2.437
Rearfoot pressure, left	6.14	8.21	+2.481	−0.584–4.979
Rearfoot pressure, right	6.36	8.52	+2.067	0.420–3.594

Note. LP = linear programming; pp = CV percentage points. Adjustment includes age, sex, and BMI plus an intercept. Percentile intervals use 1000 participant-pairs resamples per endpoint and are not multiplicity-adjusted. There are no significance stars or FDR discovery claims. Historical fitted-model results and current numerical sensitivity are separated in Supplementary Tables S5, S12 and S13.

## Data Availability

The third-party source files are accessible through AI Hub as Musculoskeletal Disease Biosignal Data, dataset 71966, version 1.1 [14], subject to platform access rules and terms of use. The authors do not redistribute the original JSON files or participant-level derived matrices. Supplementary Files S1 and S2 provide aggregate results, analytical specifications, code, seeds, and numerical-audit documentation. No new public-repository deposit or DOI is claimed. The supplied code requires authorized source data for participant-level reproduction; aggregate-only checks do not substitute for that reproduction.

## Supplementary Materials

The following supporting information can be downloaded at: https://www.mdpi.com/article/10.3390/s26206519/s1, Supplementary File S1 contains the original analysis-provenance statement, cohort and historical aggregate results, and expanded Tables S1–S14 for distributional, ICC, absolute-variability, empirical-averaging, temporal, scale, regression, numerical-sensitivity, and categorical analyses. Supplementary File S2 contains executed analytical code with version/status notes, full-precision aggregate reporting files, and the figure-generation script. Four revised figures are supplied separately as 600-dpi PNG/TIFF and vector PDF. Participant-level matrices and original AI Hub JSON files are not redistributed.
