# Supplementary material for: Temporal Consistency and Averaging Sensitivity of Released Smart-Insole Gait Summaries in Participants with and without Knee Osteoarthritis
Source: Sensors (Basel). 2026 Oct 14;26(20):6519. doi: 10.3390/s26206519 (PMC13708872; doi:10.3390/s26206519)
Supplement: Supplementary file 1 [file sensors-26-06519-s001.zip › Supplementary File S1.pdf]

Supplementary File S1

Analysis provenance and reading guide

The submitted manuscript records formalization on 2 August 2026 after feasibility exploration, without prospective registration. Post-review additions were performed on 28 September 2026 and are not represented as prespecified in that original plan. Tables S1–S5 retain or identify original aggregate results for traceability; Tables S6–S14 provide the post-review diagnostics, repaired regression summaries, and relocated categorical results. A10 is model-based. Current regression intervals are exploratory and unadjusted for multiplicity. The original six-FDR-discovery statement does not describe them.

The analysis uses 178 participants with 10 released indices each, after exclusion of the previously reported opposing-label pair. Calendar dates, original 15-day completeness, exposure duration, device calibration, and criterion measures are unavailable. Source JSONs, participant matrices, and participant-level covariates are not redistributed. Supplementary File S2 contains code and full-precision aggregate reporting data. The retained automatic numerical review status is not converted to a blanket statistical-validation PASS.

Table S1. Original characteristics of the primary cohort.

Group	*n*	Women, *n* (%)	Age, years	BMI, kg/m²
Normal	89	59 (66.3)	50.0 ± 8.7	23.0 ± 3.4
Knee OA	89	73 (82.0)	58.6 ± 9.7	24.4 ± 3.7
Total	178	132 (74.2)	54.3 ± 10.1	23.7 ± 3.6

Note. Groups were not matched on age or sex. Values are retained from the submitted aggregate cohort table.

Table S2. Original normalized variance-component display and model-based ICC(A,10).

Summary	Subject (%)	Occasion (%)	Residual (%)	ICC(A,10), original percentile CI
Balance, left	1.38	0.034	98.59	0.123 (−0.201–0.373)
Balance, right	1.43	0.039	98.53	0.127 (−0.199–0.378)
Stride length, left	66.29	0.001	33.71	0.952 (0.936–0.964)
Stride length, right	66.40	0.000	33.60	0.952 (0.934–0.966)
Gait distance	39.80	0.000	60.20	0.869 (0.812–0.913)
Gait speed	71.63	0.007	28.36	0.962 (0.951–0.970)
Forefoot pressure, left	69.18	0.087	30.73	0.957 (0.940–0.970)
Forefoot pressure, right	72.66	0.000	27.34	0.964 (0.945–0.976)
Midfoot pressure, left	84.45	0.018	15.54	0.982 (0.975–0.987)
Midfoot pressure, right	83.27	0.004	16.73	0.980 (0.973–0.985)
Rearfoot pressure, left	82.68	0.110	17.21	0.979 (0.967–0.986)
Rearfoot pressure, right	83.50	0.047	16.45	0.981 (0.965–0.989)

Note. The original display applies max(component,0) to subject and occasion estimates before normalization; it is not REML. Raw ANOVA ICCs are not zero-truncated. A small common occasion component does not demonstrate an absence of participant-specific variation or serial dependence. ICC(A,10) is not an empirical comparison of two independent 10-observation averages.

Table S3. Original descriptive group-specific ICCs with percentile intervals.

Summary	Group	ICC(A,1), 95% CI	Model ICC(A,10), 95% CI
Balance, left	Normal	0.078 (0.030–0.173)	0.460 (0.236–0.677)
Balance, left	OA	0.000 (−0.032–0.045)	0.003 (−0.447–0.320)
Balance, right	Normal	0.078 (0.030–0.172)	0.460 (0.235–0.675)
Balance, right	OA	0.001 (−0.031–0.047)	0.009 (−0.438–0.331)
Stride length, left	Normal	0.772 (0.686–0.844)	0.971 (0.956–0.982)

Summary	Group	ICC(A,1), 95% CI	Model ICC(A,10), 95% CI
Stride length, left	OA	0.532 (0.401–0.644)	0.919 (0.870–0.948)
Stride length, right	Normal	0.713 (0.606–0.813)	0.961 (0.939–0.977)
Stride length, right	OA	0.558 (0.418–0.677)	0.927 (0.878–0.955)
Gait distance	Normal	0.356 (0.224–0.490)	0.847 (0.743–0.906)
Gait distance	OA	0.404 (0.285–0.582)	0.871 (0.799–0.933)
Gait speed	Normal	0.722 (0.649–0.776)	0.963 (0.949–0.972)
Gait speed	OA	0.643 (0.529–0.730)	0.947 (0.918–0.964)
Forefoot pressure, left	Normal	0.811 (0.709–0.883)	0.977 (0.961–0.987)
Forefoot pressure, left	OA	0.622 (0.497–0.720)	0.943 (0.908–0.963)
Forefoot pressure, right	Normal	0.804 (0.697–0.878)	0.976 (0.958–0.986)
Forefoot pressure, right	OA	0.664 (0.498–0.788)	0.952 (0.908–0.974)
Midfoot pressure, left	Normal	0.899 (0.847–0.934)	0.989 (0.982–0.993)
Midfoot pressure, left	OA	0.799 (0.712–0.858)	0.975 (0.961–0.984)
Midfoot pressure, right	Normal	0.888 (0.841–0.920)	0.987 (0.981–0.991)
Midfoot pressure, right	OA	0.771 (0.680–0.838)	0.971 (0.955–0.981)
Rearfoot pressure, left	Normal	0.813 (0.665–0.907)	0.978 (0.952–0.990)
Rearfoot pressure, left	OA	0.834 (0.751–0.876)	0.981 (0.968–0.986)
Rearfoot pressure, right	Normal	0.814 (0.698–0.891)	0.978 (0.959–0.988)
Rearfoot pressure, right	OA	0.846 (0.696–0.915)	0.982 (0.958–0.991)

Note. Each group contains 89 participants. Original uncertainty used 10,000 participant resamples within group. The earlier group-specific duration columns are omitted to avoid population-specific prescriptions. These are descriptive contrasts, not formal between-ICC tests; right balance is near-complementary.

Table S4. Original duplicate-inclusion sensitivity.

Summary	Primary ICC(A,1)	All-record ICC(A,1)	Difference
Balance, left	0.014	0.014	0.000082
Balance, right	0.014	0.014	0.000080
Stride length, left	0.663	0.661	−0.002335
Stride length, right	0.664	0.662	−0.001944
Gait distance	0.398	0.398	−0.000133
Gait speed	0.716	0.714	−0.002446
Forefoot pressure, left	0.692	0.691	−0.000680
Forefoot pressure, right	0.727	0.727	−0.000090
Midfoot pressure, left	0.844	0.844	−0.000110
Midfoot pressure, right	0.833	0.833	0.000062
Rearfoot pressure, left	0.827	0.826	−0.001009
Rearfoot pressure, right	0.835	0.833	−0.002134

Note. The original analysis restored the two excluded records (n = 180 versus 178). ICC point estimates use three decimals, as in main Table 1; differences retain six decimals to show small changes calculated from unrounded values. The maximum absolute change was approximately 0.00245. This is not a test of unobserved selection or device validity.

Table S5. Historical submitted fitted-model CV results, retained for comparison only.

Summary	Original difference (pp)	Original model CI	Original p	Original BH q
Balance, left	+0.335	−0.063–0.732	0.0985	0.1460

Summary	Original difference (pp)	Original model CI	Original p	Original BH q
Balance, right	+0.367	−0.035–0.769	0.0733	0.1256
Stride length, left	+2.050	0.830–3.269	0.0011	0.0067
Stride length, right	+1.317	0.207–2.426	0.0203	0.0489
Gait distance	+0.172	−4.239–4.583	0.9388	0.9388
Gait speed	+3.610	1.787–5.433	0.0001	0.0016
Forefoot pressure, left	+2.190	0.285–4.095	0.0245	0.0489
Forefoot pressure, right	+1.111	−0.491–2.713	0.1727	0.1884
Midfoot pressure, left	+1.301	−0.295–2.898	0.1095	0.1460
Midfoot pressure, right	+1.000	−0.373–2.373	0.1522	0.1826
Rearfoot pressure, left	+2.481	0.332–4.629	0.0239	0.0489
Rearfoot pressure, right	+2.067	0.301–3.833	0.0221	0.0489

Note. These values describe the submitted model-based analysis and original 12-test family. They are not current LP bootstrap p values and are not used to claim post-review discoveries. The current estimates and nominal intervals are in Table 4/S12. Retention documents the analysis change rather than selecting results according to significance.

Table S6. Post-review distribution and IQR-flag summaries.

Summary	Range	Q1 / median / Q3	Skewness	IQR flags, *n* (%)
Balance, left	29.0 to 69.0	50.0 / 50.0 / 50.0	−0.846	681 (38.26%)
Balance, right	31.0 to 71.0	50.0 / 50.0 / 50.0	0.918	681 (38.26%)
Stride length, left	50.0 to 175.0	119.0 / 128.0 / 137.0	−0.743	36 (2.02%)
Stride length, right	55.0 to 171.0	119.0 / 127.5 / 137.0	−0.585	41 (2.30%)
Gait distance	22.0 to 10160.0	1490.0 / 1740.0 / 2050.0	3.205	102 (5.73%)
Gait speed	1.4 to 6.5	3.8 / 4.3 / 4.8	−0.434	18 (1.01%)
Forefoot pressure, left	8.0 to 77.0	31.0 / 36.0 / 40.0	0.503	61 (3.43%)
Forefoot pressure, right	3.0 to 80.0	33.0 / 37.0 / 41.0	0.215	81 (4.55%)
Midfoot pressure, left	9.0 to 56.0	31.0 / 36.0 / 40.0	−0.362	15 (0.84%)
Midfoot pressure, right	7.0 to 52.0	31.0 / 36.0 / 40.0	−0.575	18 (1.01%)
Rearfoot pressure, left	8.0 to 80.0	23.0 / 28.0 / 33.0	1.231	25 (1.40%)
Rearfoot pressure, right	5.0 to 90.0	23.0 / 27.0 / 31.0	1.871	51 (2.87%)

Note. Every field has 1780 finite observations and missing_n = 0 in the retained release. Flags use 1.5 × IQR fences and were not exclusions. Balance IQR = 0, so every value differing from 50 is flagged; this does not classify 681 observations as invalid. Units remain the released scales.

Table S7. Same-resample percentile and BCa ICC(A,1) intervals.

Summary	ICC(A,1)	Percentile 95% CI	BCa 95% CI
Balance, left	0.014	−0.017–0.056	−0.019–0.052
Balance, right	0.014	−0.017–0.057	−0.019–0.053
Stride length, left	0.663	0.593–0.730	0.590–0.729
Stride length, right	0.664	0.585–0.737	0.587–0.739
Gait distance	0.398	0.301–0.512	0.289–0.496
Gait speed	0.716	0.658–0.766	0.662–0.768
Forefoot pressure, left	0.692	0.610–0.765	0.613–0.767
Forefoot pressure, right	0.727	0.632–0.803	0.641–0.812
Midfoot pressure, left	0.844	0.797–0.881	0.797–0.881

Summary	ICC(A,1)	Percentile 95% CI	BCa 95% CI
Midfoot pressure, right	0.833	0.785–0.871	0.784–0.871
Rearfoot pressure, left	0.827	0.748–0.874	0.739–0.872
Rearfoot pressure, right	0.835	0.736–0.897	0.761–0.912

Note. 10,000 participant resamples preserve all 10 observations per selected participant. Base seed 20260902 + j, j = 0,…,11 in this table's order. Leave-one-participant-out jackknife estimates BCa acceleration. Unconstrained ICC limits are not truncated at zero; no empirical coverage study was performed.

Table S8. Scale-dependent residual variability and adjacent-difference bands.

Summary	Mean	Residual SD	SD/|mean| (%)	Nominal RC95	Descriptive band
Balance, left	49.874	2.388	4.79	6.618	−6.621–6.663
Balance, right	50.128	2.387	4.76	6.618	−6.668–6.627
Stride length, left	127.598	8.655	6.78	23.990	−22.882–23.074
Stride length, right	127.302	8.467	6.65	23.470	−22.472–22.265
Gait distance	1847.194	513.679	27.81	1423.844	−1400.278–1384.715
Gait speed	4.251	0.398	9.36	1.103	−1.035–1.035
Forefoot pressure, left	36.141	4.341	12.01	12.032	−11.508–11.311
Forefoot pressure, right	37.245	3.999	10.74	11.085	−10.103–10.147
Midfoot pressure, left	35.321	2.706	7.66	7.500	−6.714–6.769
Midfoot pressure, right	34.931	2.779	7.96	7.703	−6.769–6.759
Rearfoot pressure, left	28.538	3.273	11.47	9.072	−7.941–8.083
Rearfoot pressure, right	27.824	3.091	11.11	8.569	−7.410–7.376

Note. SD = $\sqrt{MSE}$, excluding the common occasion component. RC95 = $1.96\sqrt{2} \times SD$; the code's MDC95 and repeatability columns are identical. Bands use correlated adjacent differences and are not formal repeated-measures Bland–Altman coverage estimates. Means and scale-dependent quantities must not be treated as calibrated device-error or clinical-change thresholds.

Table S9. Complete empirical-versus-model averaging results.

Summary	m	Blocks	Predicted	Empirical	E − P
Balance, left	2	5	0.027167	0.034337	+0.007171
Balance, left	3	3	0.040204	0.047502	+0.007298
Balance, left	5	2	0.065257	0.042746	−0.022511
Stride length, left	2	5	0.797297	0.790166	−0.007131
Stride length, left	3	3	0.855072	0.822005	−0.033067
Stride length, left	5	2	0.907692	0.870440	−0.037252
Stride length, right	2	5	0.798177	0.786898	−0.011279
Stride length, right	3	3	0.855747	0.854675	−0.001072
Stride length, right	5	2	0.908148	0.896105	−0.012044
Gait distance	2	5	0.569481	0.557989	−0.011492
Gait distance	3	3	0.664898	0.632441	−0.032457
Gait distance	5	2	0.767817	0.725038	−0.042779
Gait speed	2	5	0.834714	0.824048	−0.010667
Gait speed	3	3	0.883385	0.850365	−0.033020
Gait speed	5	2	0.926607	0.886521	−0.040087
Forefoot pressure, left	2	5	0.817861	0.800273	−0.017588
Forefoot pressure, left	3	3	0.870725	0.838809	−0.031917

Summary	m	Blocks	Predicted	Empirical	E − P
Forefoot pressure, left	5	2	0.918206	0.879396	−0.038809
Forefoot pressure, right	2	5	0.842061	0.812061	−0.030000
Forefoot pressure, right	3	3	0.888856	0.863652	−0.025204
Forefoot pressure, right	5	2	0.930211	0.906718	−0.023493
Midfoot pressure, left	2	5	0.915666	0.900576	−0.015091
Midfoot pressure, left	3	3	0.942151	0.925022	−0.017130
Midfoot pressure, left	5	2	0.964469	0.936475	−0.027994
Midfoot pressure, right	2	5	0.908692	0.887521	−0.021171
Midfoot pressure, right	3	3	0.937217	0.913091	−0.024126
Midfoot pressure, right	5	2	0.961360	0.938888	−0.022472
Rearfoot pressure, left	2	5	0.905216	0.874529	−0.030687
Rearfoot pressure, left	3	3	0.934749	0.918908	−0.015841
Rearfoot pressure, left	5	2	0.959800	0.941269	−0.018531
Rearfoot pressure, right	2	5	0.910071	0.878214	−0.031857
Rearfoot pressure, right	3	3	0.938195	0.922976	−0.015219
Rearfoot pressure, right	5	2	0.961977	0.930884	−0.031092

Note. m = 2 uses indices 1–10; m = 3 uses 1–9; m = 5 uses 1–10. No second disjoint m = 10 average exists. Nonoverlap does not imply independence. Differences are descriptive and have no calculated inferential interval.

Table S10. Released-index diagnostics, not calendar-time validation.

Summary	Slope/index	Cluster SE	Trend p / BH q	Centered lag-1 r (nominal CI)
Balance, left	0.0153	0.0183	0.4029 / 0.6594	−0.127 (−0.214–−0.053)
Stride length, left	0.0859	0.0854	0.3147 / 0.6594	−0.022 (−0.063–0.026)
Stride length, right	−0.0237	0.0807	0.7693 / 0.8462	−0.067 (−0.119–−0.015)
Gait distance	−7.0783	5.0359	0.1598 / 0.5861	−0.090 (−0.245–0.098)
Gait speed	0.0016	0.0043	0.7131 / 0.8462	−0.004 (−0.065–0.054)
Forefoot pressure, left	−0.0733	0.0441	0.0967 / 0.5317	0.016 (−0.042–0.088)
Forefoot pressure, right	−0.0046	0.0419	0.9133 / 0.9133	0.035 (−0.031–0.116)
Midfoot pressure, left	−0.0093	0.0307	0.7614 / 0.8462	0.090 (0.012–0.172)
Midfoot pressure, right	−0.0269	0.0334	0.4196 / 0.6594	0.091 (0.014–0.177)
Rearfoot pressure, left	0.0826	0.0375	0.0274 / 0.3010	0.125 (0.006–0.249)
Rearfoot pressure, right	0.0315	0.0356	0.3760 / 0.6594	0.147 (0.033–0.253)

Note. Trends regress participant-centered values on centered released index with participant-cluster-robust SE; BH is across 11 trends. Lag intervals use 2000 participant resamples (base + 100 + j). Finite-series centering can induce dependence; negative r is not evidence of physiological negative autocorrelation. Calendar spacing, weekday, adaptation, and wear-time effects are unresolved. No AR(1)-adjusted projection was fitted.

Table S11. Gait-distance scale sensitivity.

Scale	ICC(A,1)	Model ICC(A,10)	Skewness
raw	0.398094	0.868661	3.205367
log1p	0.472254	0.899482	−1.028234

Note. log1p = log(1 + distance), using all observations. This changes the estimand's scale; the transformed results do not replace the original-scale analysis or prove normally distributed data.

Table S12. Current LP class coefficients and participant-bootstrap uncertainty.

Summary	Coefficient (pp)	Nominal 95% CI (pp)	Bootstrap SD (pp)	RNG seed
Balance, left	+0.334540	0.014004–1.029696	0.260112	20261102
Stride length, left	+2.049526	0.642785–3.953691	0.828557	20261103
Stride length, right	+1.316557	0.468034–2.690567	0.534172	20261104
Gait distance	+0.171982	−3.344588–5.880212	2.201008	20261105
Gait speed	+3.609798	1.628479–6.400251	1.234502	20261106
Forefoot pressure, left	+2.183419	0.052323–4.242822	1.073217	20261107
Forefoot pressure, right	+1.111266	−0.726614–2.004481	0.729028	20261108
Midfoot pressure, left	+1.301330	−0.346006–3.526022	1.024166	20261109
Midfoot pressure, right	+1.000374	−0.613734–2.436956	0.783960	20261110
Rearfoot pressure, left	+2.480678	−0.584255–4.979468	1.493260	20261111
Rearfoot pressure, right	+2.066990	0.419815–3.594464	0.805632	20261112

Note. Unpenalized conditional-median regression includes class, age, sex, BMI, and an intercept. HiGHS dual simplex is the designated reference; 1000 pairs-bootstrap draws per endpoint are retained. RNG seeds are integers: 20260902 + 200 + j, where j = 0,…,10 in table order. The resulting values 20261102–20261112 are not calendar dates or analysis dates. No p values, significance stars, or adjusted-discovery labels are assigned to these intervals. Left rearfoot CI spans zero, and left forefoot coefficient changed relative to the original iterative fit.

Table S13. Numerical coefficient-choice sensitivity of current LP bootstrap intervals.

Summary	Range-screen draws	Tolerance-sensitive draws	Lower-end span (pp)	Upper-end span (pp)	Max reference shift (pp)
Balance, left	223	10	0.0095	0.0118	0.0118
Stride length, left	172	9	0.0433	0.0537	0.0385
Stride length, right	194	15	0.0000	0.0418	0.0418
Gait distance	154	41	0.0622	0.0190	0.0551
Gait speed	185	25	0.1311	0.0995	0.0996
Forefoot pressure, left	208	18	0.0237	0.0786	0.0786
Forefoot pressure, right	176	16	0.0218	0.0429	0.0429
Midfoot pressure, left	169	11	0.0720	0.0481	0.0719
Midfoot pressure, right	178	15	0.0422	0.0333	0.0422
Rearfoot pressure, left	157	21	0.0777	0.0000	0.0777
Rearfoot pressure, right	155	18	0.0023	0.0067	0.0067

Note. Counts are not fit failures. Tight loss budget: $L^* + 10^{-10}\max(1,L^*)$ in the scaled problem; numerical screen: 0.0001 pp. A 10^{-8} budget was checked only for the 1971 screen-positive draws, not all 11,000. There were 199 tolerance-sensitive draws; no full-data coefficient range exceeded the screen. The maximum full-data range was 0.00000364758 pp. The original status SOLUTION_TOLERANCE_REVIEW_REQUIRED is retained. Within audited tight-budget choices, zero-inclusion was unchanged in 11/11 intervals; this is not coverage or FDR validation. Full bounds, not replacement CIs, appear in S2.

Table S14. Original nominal foot-angle label persistence.

Label	Fleiss κ	Exact (%)	Modal (%)	Normal exact (%)	OA exact (%)
Foot-angle category, left	0.694	94.4	97.9	94.4	94.4
Foot-angle category, right	0.625	89.3	97.1	91.0	87.6

Note. Exact = same provider label over all 10 observations; modal = mean participant modal-category agreement. Codes 0/1/2 remain nominal. These summaries cannot reveal continuous angle drift, proximity to thresholds, device-angle accuracy, or clinical-classification stability.

Supplementary File S2: reproducibility scope

S2 distinguishes original aggregate comparators, post-review diagnostics, current LP reporting outputs, and executed code. The original post-review code contains a superseded IRLS-bootstrap section; its first regression output and figure must not be used as the current results. The LP repair and numerical sensitivity scripts supersede that branch. Source code is retained unaltered for provenance, with the portable reporting script generating the four current figures from curated aggregate CSVs. Stand-alone participant-level reproduction requires authorized AI Hub files; the source matrix is not included.

No REML/G-theory benchmark, independent empirical 10-observation comparison, formal coverage study, criterion-device validation, or clinically anchored MDC analysis is represented as completed. The local seed, finite-data checks, numerical certificates, and aggregate arithmetic checks address reproducibility and numerical consistency, not those missing forms of validation.
